# Reduced field-of-view stack-of-spirals enables high spatiotemporal resolution 3D perfusion imaging

**DOI:** 10.1186/1532-429X-18-S1-P325

**Published:** 2016-01-27

**Authors:** Yang Yang, Li Zhao, Xiao Chen, Kelvin Chow, Peter W Shaw, Jorge A Gonzalez, Frederick H Epstein, Craig H Meyer, Christopher M Kramer, Michael Salerno

**Affiliations:** 1 grid.27755.32000000009136933XBiomedical Engineering, University of Virginia, Charlottesville, VA USA; 2 grid.27755.32000000009136933XMedicine, University of Virginia, Charlottesville, VA USA; 3 grid.27755.32000000009136933XRadiology, University of Virginia, Charlottesville, VA USA; 4 Siemens Medical Solutions, Princeton, NJ USA; 5 grid.239395.70000000090118547Beth Israel Deaconess Medical Center, Harvard Medical School, Boston, MA USA

## Background

First-pass contrast-enhanced myocardial perfusion CMR is a useful non-invasive technique for evaluating coronary artery disease. 3D perfusion imaging provides images at the same cardiac phase which may be advantageous for quantifying ischemic burden, but current techniques have limited spatiotemporal resolution. We previously presented a 3D stack-of-spiral (SoS) perfusion method, however this technique had a long temporal footprint (240 ms) and thick slices (8 mm). As the heart occupies only a small portion of the chest, reduced FOV (rFOV) techniques enable imaging over a small FOV with substantially improved sampling efficiency. We recently demonstrated a 2D outer volume suppressed (OVS) single-shot spiral perfusion sequence with a temporal resolution of 8 ms per image and whole heart coverage with 2 mm resolution. We hypothesized that application of OVS to 3D SoS perfusion imaging could reduce the temporal footprint while increasing the through-plane spatial resolution.

## Methods

**Figure 1:**
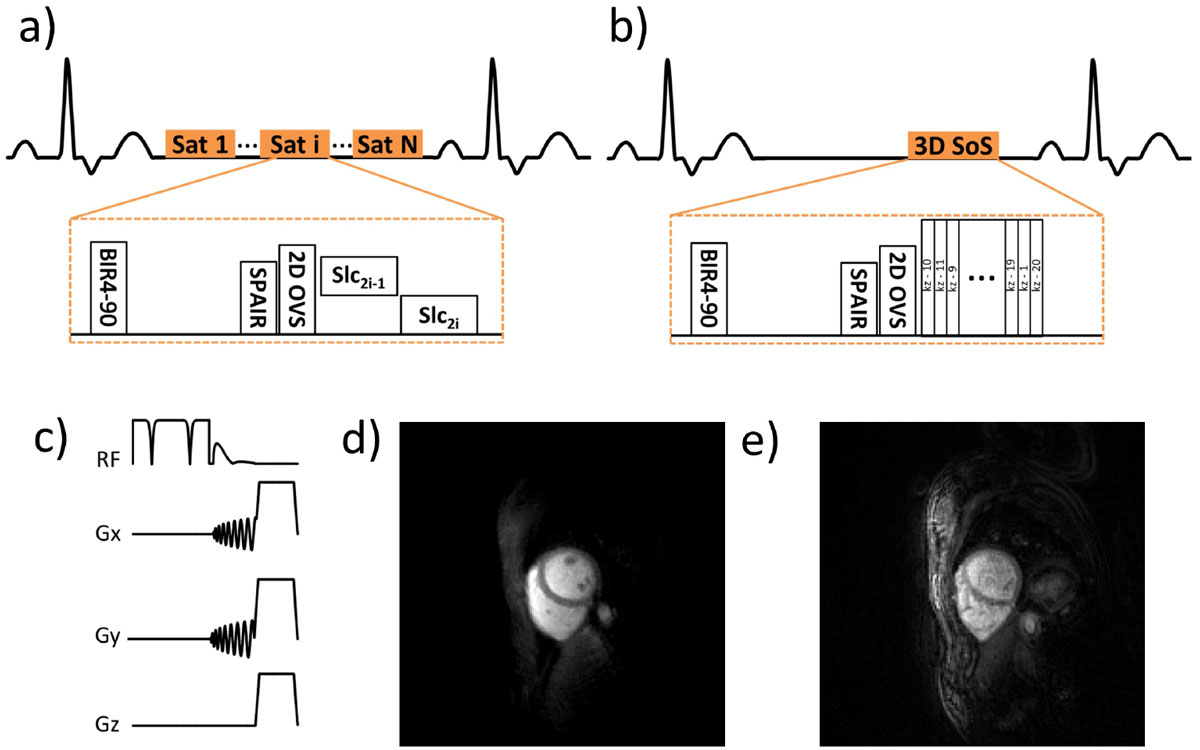
**a) 2D single-shot spiral perfusion sequence with outer volume suppression (OVS)**. b) 3D stack-of-spirals (SoS) perfusion sequence with OVS and centric ordering. c) OVS preparation consisted of a BIR4 tip-down, a 2D spiral tip-back and a spoiler. 2D (d) and 3D (e) OVS perfusion images show attenuation of signal outside of the heart region.

An OVS preparation was incorporated into a 2D single-shot (Figure 1a) and 3D SoS (Figure 1b) perfusion sequences. The OVS consisted of a non-selective adiabatic BIR-4 tip-down pulse, a 2D spiral spatially selective tip-back pulse and a spoiler to suppress signal outside the heart (Figure 1c). 2D and 3D first-pass perfusion were performed with a 0.075 mmol/kg Gd-DTPA bolus, separated by 20 min contrast washout time, in 6 subjects on a 1.5T Avanto Siemens scanner. 2D sequence parameters included: FOV 170 mm, TE 1.0 ms, TR 9 ms, SRT 80 ms, FA 90^o^, 8 ms per slice, 8 slices with 8 mm thickness, 2 mm^2^ in-plane resolution. 3D sequence parameters were similar except: SRT 150 ms, FA 35^o^, acquisition time 180 ms, 20 slices with 4 mm thickness. The images were reconstructed using Block LOw-rank Sparsity with Motion guidance (BLOSM) combined with SENSE.

## Results

**Figure 2:**
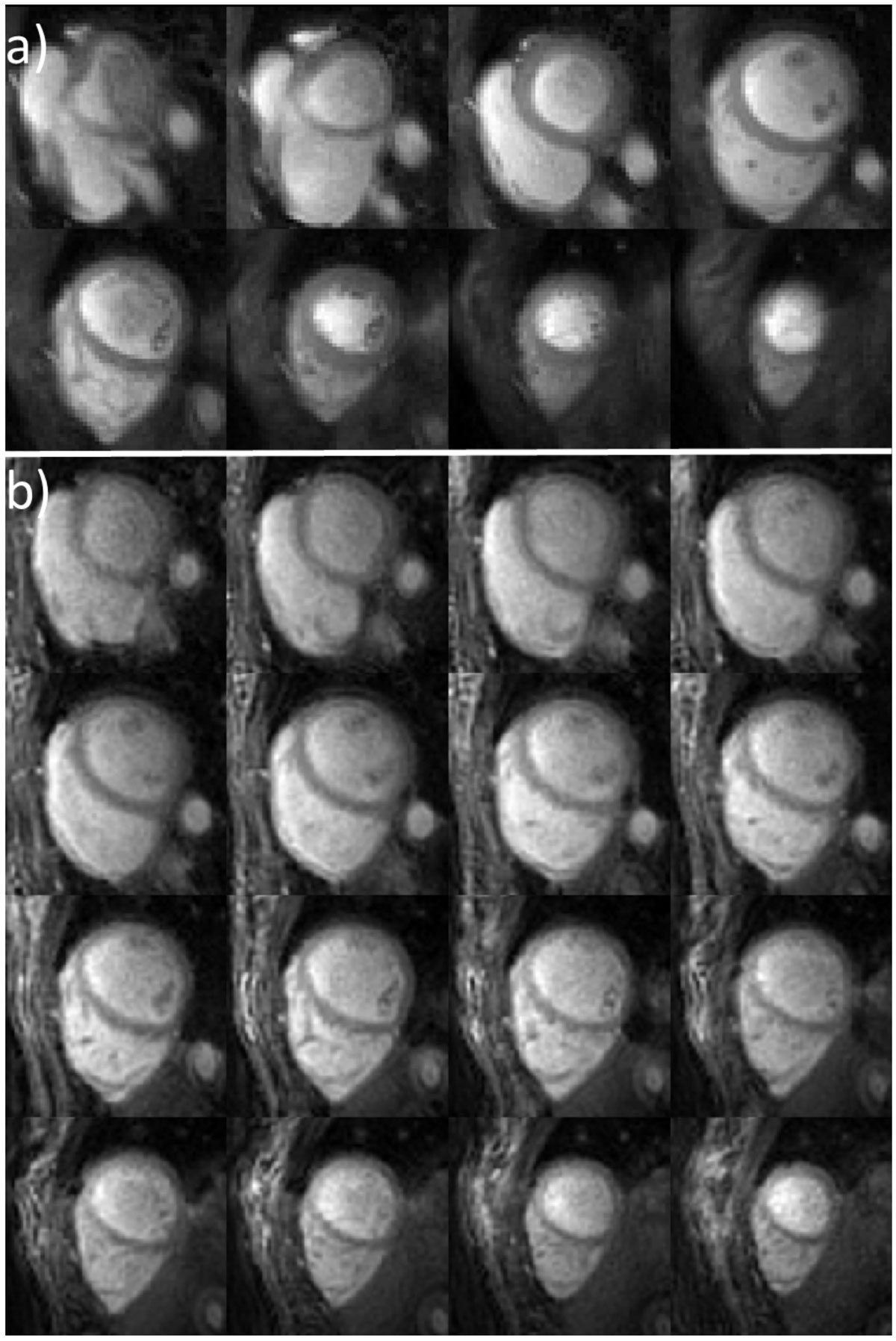
**Rest perfusion images with whole heart covearge at middle time frame from the same subject using the 2D single-shot spiral perfusion sequence with OVS (a) and 3D stack-of-spirals sequence with OVS (b)**.

Better outer volume suppression was observed for the 2D technique as compared to the 3D technique (Figure 1d-e) due to its shorter acquisition time. However, in both cases, OVS significantly attenuated signal outside of the heart, reducing spatial aliasing artifacts for both 2D and 3D techniques. Figure 2 shows 2D single-shot and 3D SoS perfusion images from the same subject at a similar time point. Both the 2D and 3D techniques resulted in good image quality. Given the very short temporal footprint of the 2D single-shot technique, fine details of the cardiac trabeculae and papillary muscles are better resolved than with the 3D technique. 3D SoS provided high through-plane resolution (4 mm) reducing partial volume effects and provided better depiction of the apical slices, but with some loss of fine detail due to the longer temporal footprint. Similar image quality was found for the other cases.

## Conclusions

We demonstrated the successful application of OVS to 3D SoS perfusion techniques. The improvement of sampling efficiency using OVS enables 3D imaging with a combination of high in-plane and through-plane spatial resolution and a temporal footprint of 180 ms.

